# Rapid Weight Loss and Severe Failure to Thrive Mimicking Lipodystrophy Syndrome in a 1-Year-Old Taiwanese Girl with Costello Syndrome

**DOI:** 10.3390/children9060905

**Published:** 2022-06-16

**Authors:** Yu-Min Syu, Hung-Chang Lee, Jui-Hsing Chang, Chung-Lin Lee, Chih-Kuang Chuang, Huei-Ching Chiu, Ya-Hui Chang, Hsiang-Yu Lin, Shuan-Pei Lin

**Affiliations:** 1 Department of Pediatrics, Far Eastern Memorial Hospital, New Taipei City 22021, Taiwan; b101098040@tmu.edu.tw; 2 Division of Genetics and Metabolism, Department of Pediatrics, MacKay Memorial Hospital, Taipei 10449, Taiwan; clampcage@yahoo.com.tw (C.-L.L.); g880a01@mmh.org.tw (H.-C.C.); wish1001026@gmail.com (Y.-H.C.); 3 Division of Gastroenterology and Nutrition, Department of Pediatrics, MacKay Children’s Hospital, Taipei 10449, Taiwan; ped2435@mmh.org.tw; 4 Department of Medicine, MacKay Medical College, New Taipei City 25245, Taiwan; jhchang90@yahoo.com.tw; 5 Department of Pediatrics, MacKay Children’s Hospital, Taipei 10449, Taiwan; 6 Department of Rare Disease Center, MacKay Memorial Hospital, Taipei 10449, Taiwan; 7 Institute of Clinical Medicine, National Yang-Ming Chiao Tung University, Taipei 11221, Taiwan; 8 Division of Genetics and Metabolism, Department of Medical Research, MacKay Memorial Hospital, Taipei 10449, Taiwan; mmhcck@gmail.com; 9 College of Medicine, Fu-Jen Catholic University, Taipei 24205, Taiwan; 10 Department of Childhood Care and Education, MacKay Junior College of Medicine, Nursing and Management, Taipei 11260, Taiwan; 11 Department of Medical Research, China Medical University Hospital, China Medical University, Taichung 40402, Taiwan; 12 Department of Infant and Child Care, National Taipei University of Nursing and Health Sciences, Taipei 11219, Taiwan

**Keywords:** Costello syndrome, RASopathy, failure to thrive, dysmorphism, nutritional status

## Abstract

Costello syndrome (CS) is a type of RASopathy caused mainly by de-novo heterozygous pathogenic variants in the *HRAS* gene located on chromosome 11p15.5. The phenotype of CS is characterized by prenatal overgrowth, postnatal failure to thrive, curly or sparse fine hair, coarse facial features, and multisystem involvement, including cardiovascular, endocrine, and gastroenterological disorders. We present a one-year-old girl with rapid weight loss and severe failure to thrive. She had gastroesophageal reflux at the age of four months with subsequent rapid weight loss. The loss of fat tissue over the whole body, refractory to a hypercaloric diet, mimicked the presentation of progressive lipodystrophy and masked the dysmorphic features of CS. The final diagnosis of CS was made by whole exome sequencing, which demonstrated a hot-spot, heterozygouspathogenic variant in the *HRAS* gene (c.34G > A, rs104894229). Our patient illustrates that the excessive energy needs in CS patients may lead to severe failure to thrive and cause challenges in diagnosing CS. This case also highlights the importance of recognizing CS in patients with a history of prenatal overgrowth, polyhydramnios presenting with severe failure to thrive refractory to pharmacotherapy and tube feeding.

## 1. Introduction

RASopathies are a group of rare genetic diseases caused by germline mutations in the RAS pathway genes, which play important roles in cellular growth and differentiation. Collectively, RASopathies represent one of the largest groups of multiple congenital anomaly syndromes, including Costello syndrome (CS), Noonan syndrome, Legiussyndrome, LEOPARD syndrome, neurofibromatosis1, and cardio-facio-cutaneous syndrome. These syndromeshave recognizable and overlapping phenotypic features and associated anomalies, such as cutaneous lesions, cardiac defects, and cancer susceptibility. Making an early clinical diagnosis can be challenging because of absent or uncharacteristic craniofacial features and because the complete clinical picture may evolve only with time [1,2]. Herein, we report aone-year-old girlpresenting with complicated clinical manifestations, including gastrointestinal dysfunction, rapid weight loss, and severe failure to thrive, mimicking lipodystrophy. The dysmorphic features were not distinct until her nutritional status had been optimized after the molecular diagnosis of CS, which was further affirmed by the transformation of the clinical picture.

## 2. Clinical Report

A one-year-old Taiwanese girl presented to our clinic for a second opinion due to severe failure to thrive. She was the first child of healthy non-consanguineous parents. According to previous medical records, her mother received regular prenatal examinations. The pregnancy was complicated by severe polyhydramnios, which was treated three times by amnioreduction. Amniocentesis revealed a normal female karyotype, and array comparative genomic hybridization of amniotic fluid showed negative findings. On prenatal ultrasound, polyhydramnios and increased abdominal circumference were noted. She was born at 34 weeks and 5 days of gestation by cesarean sectionat a tertiary referral center. Her birth weight was 3.18 kg (90–97th centile for gestational age), length 48 cm (50–75th centile), and occipitofrontal head circumference (OFC) 35.8 cm (>90th centile). The Apgar scores were 8 at 1 min and 9 at 5 min. She presented with general swelling and a high-arched palate, relatively large mouth, and small thoracic cavity without other distinct facial dysmorphisms. Immediately after birth, she required ventilatory support for respiratory distress and was hospitalized for 20 days. Echocardiography revealed biventricular hypertrophy with blocked premature atrial complexes, and propranolol was given thereafter. Her karyotype was normal (46,XX), and rare metabolic disorder work-up revealed negative findings. The post-discharge course was smooth, and her development was within normal limits before four months of age.

At the age of four months, she began to have diarrhea and gastroesophageal reflux (GER). Proton pump inhibitors along with prokinetic agents were prescribed, however, poor appetite, frequent regurgitation with intermittent abdominal bloating, and constipation persisted. Her body weight dropped dramatically from 4.6 kg to 3.6 kg within two months, even under medical care.

**Figure 1:**
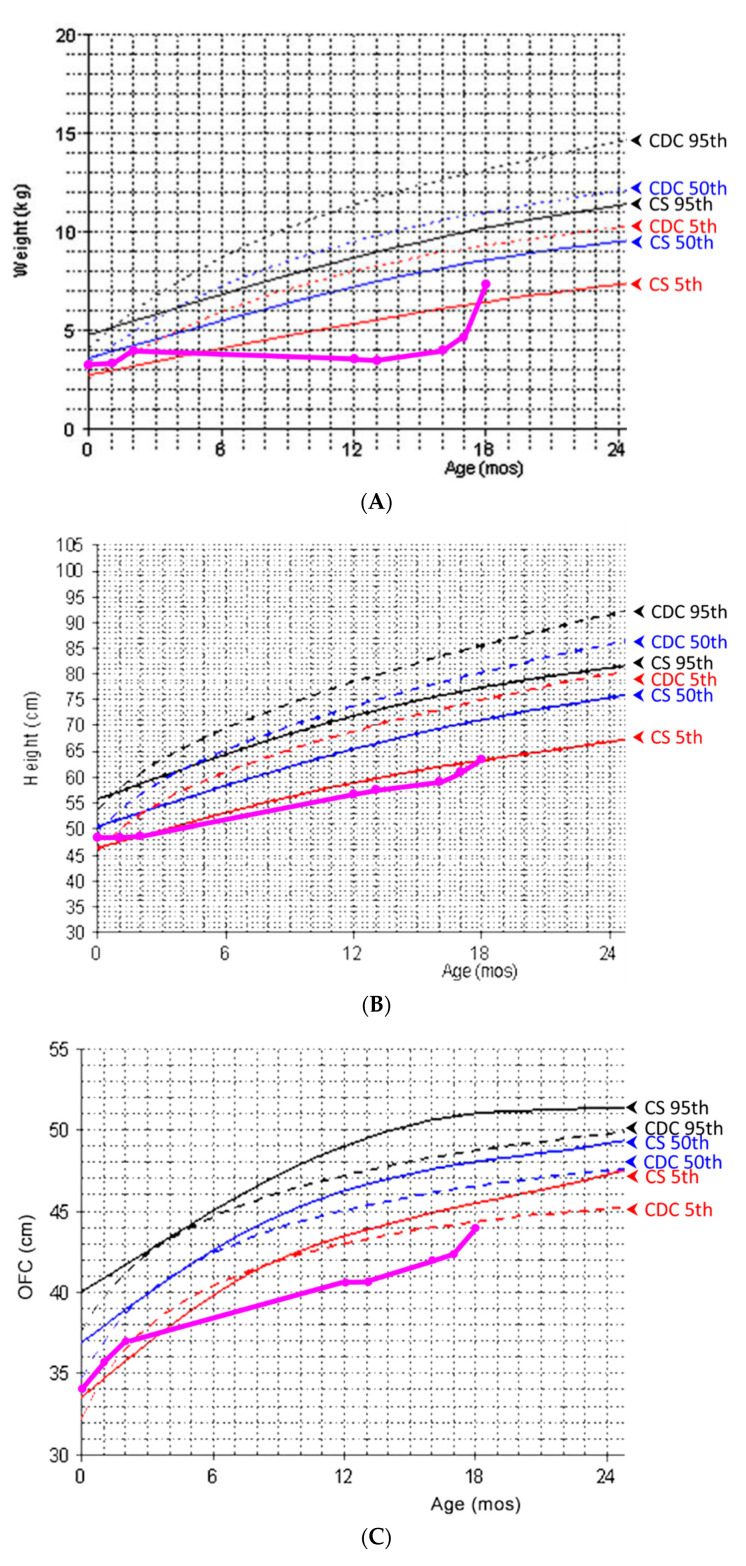
The patient’s serial body weight (**A**), length (**B**), and OFC (**C**) measurements (in pink) compared to the normative growth chart of normal individuals and CS patients. Adapted with permission from Ref. [3]. 2012, Karen W. Gripp. OFC, occipitofrontal head circumference; CDC: Centers for Disease Control and Prevention; CS: Costello syndrome.

At the time of this presentation, she was oneyear of age and weighed 3.6 kg (<3rd centile) with a body length of 56.3 cm (<3rd centile) and OFC of 40.5 cm (<3rd centile). Serial body weight, length, and OFC measurements are shown in Figure 1. Informed consent was obtained for clinical photographs and publication from her parents.

**Figure 2:**
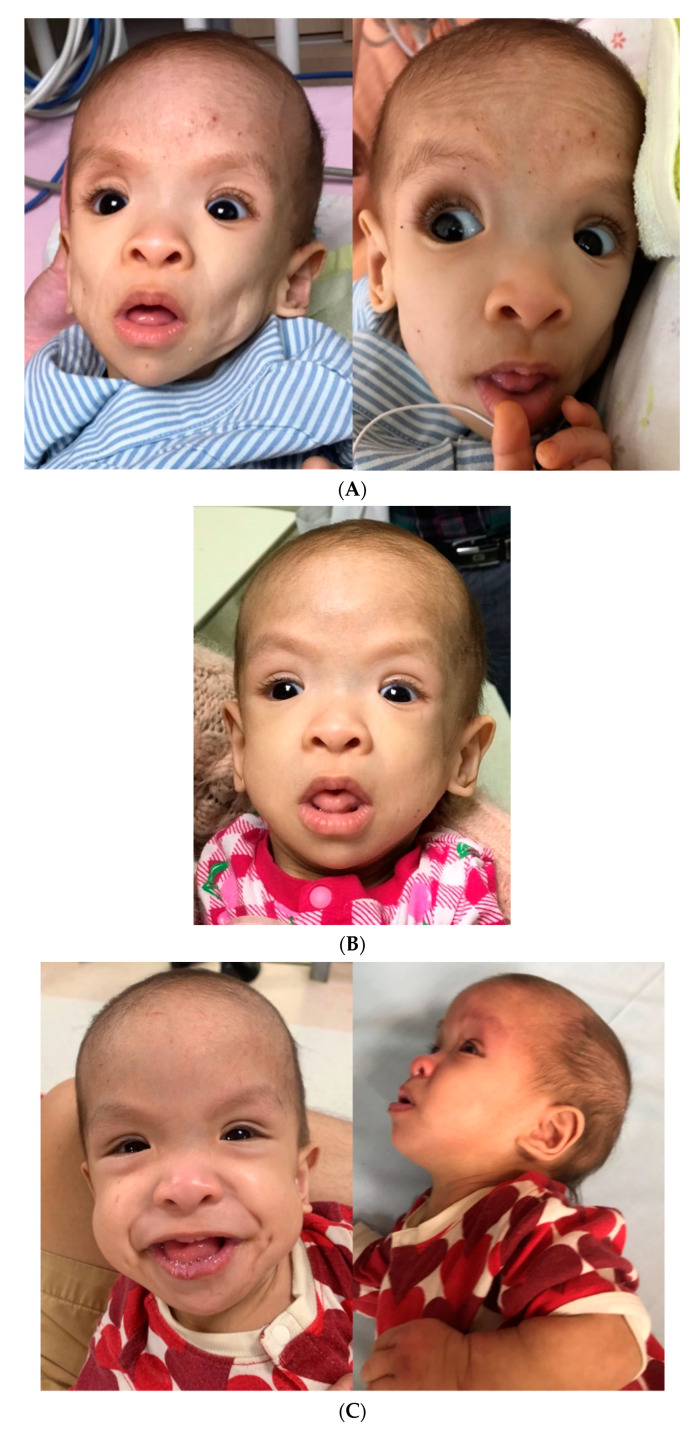
Craniofacial features of the patient. (**A**) At the age of one year, with a body weight of 3.6 kg. (**B**) At the age of one year and five months, with a body weight of 4.7 kg. Low nasal bridge and thick lips were more distinct. (**C**) At the age of one year and six months, with a body weight of 7.4 kg. Downslanting eyes, low nasal bridge, thick lips, wide nostrils and curly, sparse, thin hair were presented.

A physical examination showed a child with a severely wasting appearance and a gestalt of generalized lipodystrophy (Figure 2A). She had sparse hair, frontal bossing, deep-set eyes, relatively large mouth, skeleton-like cheeks with prominent masticatory muscles, loose and thin skin with prominent superficial veins, and very thin subcutaneous fat tissue and thin muscle layers. A neurodevelopmental examination revealed hypotonia and global delay with poor head control, absence of rolling, and language development. She had oro-motor dysfunction and was fed 450–500 kcal per day via a nasogastric tube (NG tube), corresponding to 130% of the daily calorie requirement for her ideal body weight. A cardiovascular examination, including electrocardiogram (ECG), Holter monitoring, and echocardiogram, showed a slightly thickened interventricular septum, but other findings were normal. An abdominal examination, including abdominal radiography and sonography, showed diffuse gaseousness with bowel dilatation, indicating an ileus pattern. Stool analysis results were unremarkable. A lower gastrointestinal (GI) series raised the suspicion of Hirschsprung’s disease, however, a rectal suction biopsy showed the presence of ganglion cells and staining for acetylcholinesterase was unremarkable. Her overall clinical assessment raised the suspicion of lipodystrophy. During the hospitalization, transient hypoglycemia was noted once, while her growth hormone (GH), cortisol, and insulin levels were normal. Her thyroid function, immunoglobulin profiles, lipid profiles, and metabolic investigations were also unremarkable. These findings did not indicate a syndromal diagnosis, and therefore, exome sequencing was arranged. We collected peripheral blood from the patient, and the sample was extracted by CatchGene Cell/Blood DNA Kit (CatchGene, New Taipei City, Taiwan). The whole exome sequencing was performed by IlluminaNextSeq 500 system platform with IDT xGenexome research panel v2 reagent chemistry (IDT, Coralville, IA, USA), usingNextSeq 500/550 Mid Output Kit v2.5 (300 Cycles). After sequencing was completed, the FASTQ file was uploaded to the Illuminabasespaceand processed with DRAGEN Enrichment (Illumina Inc., San Diego, CA, USA) to generate the VCF file. The variant filtering was carried out using Illuminabasespace variant interpreter. The variants were filtered by the following criterion: (1) Population frequency (in both general population and East Asian subgroup) below 0.01 in 3 different databases:The Genome Aggregation Database (http://gnomad.broadinstitute.org/), the Exome Aggregation Consortium (http://exac.broadinstitute.org/), the 1000 Genomes Project (http://browser.1000genomes.org), accessed all on 4 October 2021, (2) Sequencing depth >10 and the alternatevariant ≥ 0.25, (3) Classified as pathogenic, likely pathogenic or uncertain significances. We confirmed the identified variants via Sanger sequencing and interpreted the variants using the guidelines of the American College of Medical Genetics and Genomics (ACMG) [4].

The results of exome sequencing showed a de novo, heterozygous *HRAS* missense mutation c.34G > A (p.Gly12Ser), which we confirmed via Sanger sequencing, indicative of CS [5]. The variant was reported in ClinVar as a hot-spot mutation in *HRAS* gene and was classified as pathogenic. There was no distinct facial gestalt of CSexcept for sparse hair and relatively large mouth at the time of molecular diagnosis.

Because of the persistent GER complicated with Sandifer syndrome, laparoscopic fundoplication and gastrostomy for nutritional support were done at the age of one year and three months. A small liver with nearly no fat tissue was seen in intraoperative laparoscopy. However, the lack of weight gainpersisted after the intervention, and peripheral parenteral nutrition was, therefore, administered. With optimization of her nutritional status with peripheral parenteral nutrition, she achieved a body weight of 4.7 kg at one year and five months and 7.3 kg (5–50th centile for children with CS) at one year and six months. Downslanting eyes, low nasal bridge, thick lips, wide nostrils, and curly, sparse, thin hair developed, which were compatible with the classical dysmorphism of CS. The transformation of her craniofacial features is shown in Figure 2.

## 3. Discussion

Costello syndrome (CS) is a type of RASopathy caused mainly by de-novo heterozygous pathogenic variants in the *HRAS* gene, which is located on chromosome 11p15.5. The estimated birth prevalence of CS is 1:380,000–1,290,000 [6,7]. The phenotype of CS is characterized by prenatal polyhydramnios, overgrowth, postnatal failure to thrive, relative macrocephaly, curly or sparse fine hair, large mouth with thick lips, coarse facial features, hypotonia with developmental delay, and multisystem involvement, including cardiovascular, endocrine, and gastroenterological disorders [8,9].

Cardiovascular disease is present in 85% of individuals with CS, including hypertrophic cardiomyopathy (HCM), congenital heart defects, dysrhythmias and/or hypertension. HCM accounts for 75% of cardiovascular pathology, and the clinical course varies from a rare severe neonatal lethal form to the typical mild to moderate form of HCM seen in the majority of cases [8]. Chronic or progressive hypertrophy has been reported in 37% of affected individuals, stable disease in 25%, and improvement or resolution in 14% [10]. Given the risk of cardiovascular disorders, screening and annual follow-up with echocardiography, ECG, and Holter monitoring are warranted throughout life.

Endocrine disorders common in children with CS include hypoglycemia, GH deficiency, and problems with puberty. Neonates and infants are at high risk of hyperinsulinemichypoglycemia, while infants or young individuals often present with low serum glucose due to GH deficiency.

Feeding difficulties, poor suction power, severe GER, and failure to thrive are almost universal clinical manifestations of CS, and alternative feeding methodshave been suggested [8]. For example, ina small cohort of 20 infants with CS, 95% received NG tube placement, and 35% required gastrostomy tube (G-tube) placement. Treatment with proton pump inhibitors and prokinetic agents should be considered for gastroenterological disorders. The degree of failure to thrive is most severe at 12 months of age, and the body weight of most children with CS at that time period is below the 5th percentile compared to the general population [3]. The mean weight at one year of age has been reported to be 6.7 kg [11]. The reasons for failure to thrive are likely multifactorial, and may involve feeding difficulties, poor nutritional intake, hormonal abnormalities, increased resting energy expenditure, and dysregulated central control of body weight via leptin signaling in the hypothalamus [12,13]. Several studies have reported that CS patients follow a natural growth history in weight and height regardless of whether a normal or hypercaloric diet is given [11,13]. In addition, it has also beenreported that poor nutritional status may obscurethe classic facial gestalt of CS (downslanting eyes, low nasal bridge, full cheeks, thick lips, etc.) and cause difficulties in making a diagnosis [12].

Our patient presentedwith GI dysfunction and feeding difficulty at four months of age, both of which contributed to rapid weight loss and severe failure to thrive. Her body weight was below the 5th centile compared to the normative growth curve for CS patients [3]. Feeding by anNG tubeand then a G-tubewith adequate daily calorie supplement under medical care was tried, but the growth deficiency persisted. The disproportionate calorie needs may have indicatedincreased resting metabolism in our patient. While previous studies have documented an improvement in weight gain with either NG tube or G-tube feeding after one year of age, our patient had persistent growth deficiency, and only peripheral parenteral nutrition helped the condition. Her refractory failure to thriveand rapid loss of fat mimicked the presentation of lipodystrophy, masked the dysmorphic features of CS, and complicated the clinical picture. The low nasal bridge and coarse facial features were not obvious compared to her sunken cheeks and eyeballs. In addition, her symmetric growth deficiency with microcephaly was not compatible with the typical feature of relative macrocephaly in patients with CS. Her hypotonia and developmental delay were distinct but initially attributed to muscle wasting. The diagnosis of CS is difficult in early infancy because some characteristics may only evolve with time [1].

Besides the non-specific wasting appearance, our patient had atypical cardiovascular presentations that further complicated the diagnosis of CS. The ECG and echocardiography on presentation only showed a slightly thickened interventricular septum, but not the typical feature of HCM or arrhythmia. However, tracing back her medical history, cardiovascular disorders which may have indicated CS, such as biventricular hypertrophy and blocked premature atrial complexes, were documented after birth but resolved within one year. About 68% of CS patients with HCM have a progressive or stable clinical course, and 14% have resolution and about 70% of CS patients with atrial arrhythmias have a stable or resolved course [10]. In addition, our patient had a prenatal history of polyhydramnios and a past history of being large for gestational age with general edema, which are typical presentations of CS. A comprehensive medical record review of the cardiovascular system and pre- and postnatal history of a patient may be of value in the diagnosis of CS besides asingle evaluation at presentation.

The final diagnosis of CS was made by whole exome sequencing, which demonstrated a hot-spot, heterozygous pathogenic variant in the *HRAS* gene (c.34G > A, rs104894229). The clinical picture was not completelycompatible with the molecular diagnosis until her condition improved and the dysmorphismdeveloped over time. Our patient illustrates that excessive energy needs in CS patients may lead to severe failure to thrive that mimics the presentation of lipodystrophy and causes challenges in diagnosing CS. In infants presenting with lipodystrophicfeatures with an initial onset of GI disorders and unremarkable metabolic and lipid profiles [14], it is important for pediatricians to take CS into consideration and search for subtle clues that may be disguised by the appearance of malnutrition.Our case also highlights the importance of recognizing CS in patients with a history of prenatal overgrowth, polyhydramnios presenting with severe failure to thrive refractory to pharmacotherapy and tube feeding. The early diagnosis of CS may provide clinical insights indicating the need for alternative feeding routes or peripheral parenteral nutritional support. Optimized treatment strategies for children with CSmay lead to better development.

## Abbreviations

OFC	occipitofrontal head circumference
GER	gastroesophageal reflux
NG tube	nasogastric tube
ECG	electrocardiogram
GI	gastrointestinal
GH	growth hormone
CS	Costello syndrome
HCM	hypertrophic cardiomyopathy
G-tube	gastrostomy tube
